# Risk Factors for Acute Coronary Syndrome in Upper Gastrointestinal Bleeding Patients

**DOI:** 10.1155/2021/8816805

**Published:** 2021-03-08

**Authors:** Tianyu Chi, Quchuan Zhao, Peili Wang

**Affiliations:** ^1^Departments of Gastroenterology, Xuanwu Hospital Capital Medical University, Beijing, China; ^2^Cardiovascular Center, Xi Yuan Hospital of China Academy of Chinese Medical Sciences, Beijing, China

## Abstract

**Background:**

Upper gastrointestinal bleeding (UGIB) is a common critical disease with a certain fatality rate. Acute coronary syndrome (ACS), another critical ill condition, is a regular occurrence in the UGIB. We identified risk factors for ACS in UGIB.

**Methods:**

676 patients diagnosed with UGIB were enrolled retrospectively. We assessed the occurrence of ACS in UGIB patients and identified the risk factors for ACS by logistic regression analysis and random forest analysis.

**Results:**

After propensity score matching (PSM), the ACS group (*n* = 69) and non-ACS group (*n* = 276) were analyzed. Logistic regression analysis showed that syncope (*P* = 0.001), coronary heart disease history (*P* = 0.001), Glasgow Blatchford score (*P* ≤ 0.001), Rockall risk score (*P* = 0.004), red blood cell distribution width (RDW) (*P* ≤ 0.001), total bilirubin (TBil) (*P* = 0.046), fibrinogen (*P* ≤ 0.001), and hemoglobin (*P* = 0.001) had important roles in ACS patients. With Mean Decrease Gini (MDG) sequencing, fibrinogen, RDW, and hemoglobin were ranked the top three risk factors associated with ACS. In ROC analysis, fibrinogen (AUC = 0.841, 95% CI: 0.779-0.903) and RDW (AUC = 0.826, 95% CI: 0.769-0.883) obtained good discrimination performance. According to sensitivity > 80%, the pAUC of fibrinogen and RDW were 0.077 and 0.101, respectively, and there was no significant difference (*P* = 0.326). However, according to specificity > 80%, the pAUC of fibrinogen was higher than that of RDW (0.126 vs. 0.088, *P* = 0.018).

**Conclusion:**

Fibrinogen and RDW were important risk factors for ACS in UGIB. Additionally, combination with coronary heart disease, syncope, hemoglobin, and TBil played important roles in the occurrence of ACS. Meanwhile, it was also noted that Rockall score and Glasgow Blatchford score should be performed to predict the risk.

## 1. Introduction

Upper gastrointestinal bleeding (UGIB) is a common emergency in the internal medicine department. Despite the worldwide decrease of UGIB, incidences depend on the advanced endoscopy technology, effective drugs to suppress gastric acid, and eradication for *Helicobacter Pylori*, mortality rate of UGIB stands as high as 2-10% [1–4]. In the United States, research reported a decrease in UGIB incidence (96-82/100,000), whereas there are still nearly 300,000 cases of hospitalizations per year, with a mortality rate of around 5% [4, 5]. When UGIB is combined with acute coronary syndrome (ACS), morality rate rises to 62%, a much higher number than UGIB could generate alone (2-10%). ACS is referred as a set of progressive clinical syndromes due to critical myocardial ischemia caused by thrombus formation in coronary arteries. ACS includes unstable angina pectoris (UA), acute non-ST-segment elevation myocardial infarction (NSTEMI), and acute ST-segment elevation myocardial infarction (STEMI). It is known to all that ACS as a serious threat to human health has many complications which lead to high disability rate and mortality rate. When hemorrhage occurs in the digestive tract, blood volume in the circulation is greatly reduced, resulting in a sudden drop in cardiac output and reduced coronary artery perfusion. These changes in hemodynamics are likely to peel off the arteriosclerosis plaque which then causes the intracoronary ischemia. Studies have shown that gastrointestinal bleeding has become the most common noncardiac complication in ACS patients, while remyocardial infarction and fatality rate are significantly increased [6]. Studies also illustrate that many cases of gastrointestinal bleeding have a variety of comorbidities, or rather, many nongastrointestinal comorbidities including myocardial infarction are making up independent risk factors for gastrointestinal bleeding [7].

As people are more concerned about the signs and symptoms of severe UGIB, the disease state of UGIB and ACS is not given sufficient attention and assessment [8]. Previous diagnosis and treatment of UGIB simply observed patient's gastrointestinal symptoms and if it stopped bleeding, while it in fact needs to closely monitor the functions of other organs after UGIB, especially when UGIB is combined with ACS, both of which are clinical emergency and demand emergency treatment. Unfortunately, they run against each other in terms of treatment. There is no clear clinical instruction to follow for the prediction and treatment of UGIB combined with ACS. At present, there are few studies on the risk factors of UGIB combined with ACS, most of which only select a single biochemical index in clinical studies or only do single-factor analysis, ignoring the possibility of multiple risk factors about UGIB and ACS. Therefore, this study is a retrospective case-control study aimed at exploring the risk factors of UGIB and ACS. Dynamic monitoring of myocardial markers and electrocardiogram should be carried out in time to prevent or treat the progress of ACS as soon as possible for reducing the risk and mortality of it, when the high-risk cases with above-mentioned are detected.

## 2. Patients and Methods

### 2.1. Study Design, Setting, and Participants

We conducted a retrospective propensity score matching study to find risk factors associated with ACS in UGIB patients. The subjects were 676 patients diagnosed with UGIB in a grade A class 3 hospital in Beijing from January 2015 to January 2020, of which the experimental group was composed of patients with ACS in UGIB (*n* = 69) and the control group was made up of UGIB patients without ACS (*n* = 607). The data were collected from the hospital electronic case system. This study was approved by the Medical Ethics Committee. Inclusion criteria for the 69 cases in the experimental group included (i) Chinese nationality, (ii) outpatient onset of UGIB and hospitalized patients of UGIB, (iii) clinically and/or endoscopically verified UGIB bleeding, and (iv) ACS occurs after UGIB. The inclusion criteria for the control group included (i) Chinese nationality, (ii) outpatient onset of UGIB and hospitalized patients of UGIB, (iii) clinically and/or endoscopically verified UGIB bleeding, and (iv) no ACS after UGIB. The exclusion criteria for all subjects were (i) with incomplete medical records and (ii) those who were not coordinated with the test. All inclusion and exclusion criteria were met before the patients were enrolled.

### 2.2. Data Sources and Assessment

UGIB refers to gastrointestinal bleeding above the Treitz ligament, with ICD-10 codes including K92.204, K25.401, K25.102, K25.001, K26.601, K26.402, K26.201, K26.001, K29.002, K22.106, K92.208, I86.401, I85.901, K21.0+K22.804, C16._+K92.201, C15._+K22.804, C16.1+K92.201, K22.6, K27.4, K70.3+I98.3∗, K74.3+I98.3∗, K71.9+I98.3∗, K74.6+I98.3∗. ACS meets the diagnostic standards in the third global unified definition of myocardial infarction, with ICD-10 codes being I24.8, I21.9, I21, I20, I22, and I26. A structured form covered the following potential relative factors: demographic characteristics (gender, age, BMI), lifestyle (smoking, drinking), complications (hypertension, coronary heart disease, atrial fibrillation, cerebrovascular disease, liver cirrhosis, chronic renal disease, rheumatic disease, type 2 diabetes), history of gastrointestinal bleeding, clinical syndromes (haematemesis, melena, syncope, hemorrhagic shock), drug combination (aspirin, clopidogrel, other antiplatelet drugs, anticoagulants, glucocorticoid), the cause of upper gastrointestinal bleeding (peptic ulcer, malignant tumor, esophageal and gastric varices, Mallory-Weiss syndrome, acute gastric mucosal lesion, oesophagitis, anastomositis), interventions (mechanical ventilation, transfusion, CPR), hospital indexes ((in-hospital mortality, HLOS (hospital length of stay)), clinical scores (Glasgow-Blatchford score, Rockall score), and laboratory indicators (test indicators during the first 1-2 days after admission, including hemoglobin, RDW, blood platelet, ALT, TBil, creatinine, BUN, UA, Albumin, INR, fibrinogen, D-dimer, PaO_2_, PaCO_2_, lactic acid).

### 2.3. End Points

The primary end point was the occurrence of ACS in the patients with UGIB and to analyze the risk factors for ACS in UGIB.

### 2.4. Statistical Analysis

Categorical measurements were described as count and percentage, while continuous measurements were presented as mean and range. If continuous variables were not the normal distribution, they were described with the median and interquartile range. The statistical significance of differences was analyzed by independent *t*-test or Mann-Whitney *U* test for continuous variables and the chi-square test for categorical variables. Propensity score matching (PSM) was used by the ratio of 1 : 4 between the ACS and non-ACS groups, considering the impact of potential confounding factors and selection bias in this study. PSM variables were age and gender. Nonrandom package of R software (http://www.r-project.org) was used to implement propensity matching. After PSM was performed, the age and sex were balanced in the two matched groups. Multivariate logistic regression analysis was applied to extract risk factors of ACS, and odds ratio (OR) with 95% confidence interval (CI) of ACS was calculated. The nomogram graph was built with potential risk factors (*P* < 0.05) based on multivariate logistic analysis. The important risk factors associated with ACS were analyzed by the ROC curve. Mean Decrease Gini (MDG) involved in random forest algorithm was used to rank the important indexes with ACS. MDG provides ways to quantify which indices contribute most to classification accuracy. Greater MDG will indicate that the degree of impurity arising from the category could be reduced farthest by one variable and thus suggests an important associated index. Statistical analysis was performed using SPSS 21.0 (SPSS Inc., Chicago, IL) and random forest package of R software. All the statistical tests were two-sided and considered statistically significant if *P* < 0.05.

## 3. Results

### 3.1. Baseline Profiles

A total of 69 UGIB patients who had developed ACS were selected in the ACS group, and 607 UGIB patients without ACS were selected in the control group. The age of the ACS group was 54-82 years, with an average age being 68.52 ± 7.58; 54 patients were men (78.3%) and 15 (21.7%) were women. The age of the control group was 19-87, with an average age being 61.34 ± 14.70. 413 (68.0%) were men and 194 (32.0%) were women. The specific diseases of 69 cases of ACS were as follows: UA 20 (29.0%), NSTEMI 36 (52.2%), and STEMI 13 (18.8%). The observation indices in both groups are shown in supplement table 1.

### 3.2. Propensity Score Matching

Propensity score matching (PSM) was performed by the ratio of 1 : 4 between the ACS and non-ACS groups. PSM variables were age and gender. There were 69 cases in the ACS group and 276 cases in the control group after PSM, and then, the age and gender of the two groups were balanced (see Supplement table 2).

### 3.3. Single-Factor Analysis

The characteristics and single-factor analysis results are shown In Table 1. In ACS group, syncope (*P* ≤ 0.001), coronary heart disease (*P* ≤ 0.001), hemorrhagic shock (*P* = 0.003), in-hospital mortality (P =0.001), HLOS (P ≤0.001), Blatchford score (*P* ≤ 0.001), Rockall risk score (*P* ≤ 0.001), RDW (*P* ≤ 0.001), TBil (*P* = 0.001), UA (*P* ≤ 0.001), fibrinogen (*P* ≤ 0.001), D-dimer (*P* = 0.004), and lactic acid (*P* ≤ 0.001) were significantly higher than those in the control group. In the meantime, hemoglobin (*P* ≤ 0.001) and albumin (*P* = 0.003) in the ACS group were significantly lower than those in the control group.

### 3.4. Logistic Regression Analysis

Logistic regression analysis adjusted by the history of coronary heart disease was conducted on indicators with significant difference in single-factor analysis between the two groups to extract risk factors, and the results are shown in Table 2. The statistically significant variables are as follows: syncope (*P* = 0.001), coronary heart disease (*P* = 0.001), Blatchford score (*P* ≤ 0.001), Rockall risk score (*P* = 0.004), RDW (*P* ≤ 0.001), TBil (*P* = 0.046), fibrinogen (*P* ≤ 0.001), and hemoglobin (*P* = 0.001).

To validate the identified risk factors for ACS in UGIB, we additionally performed multiple logistic regression analysis without adjust history of coronary heart disease, and the results are shown in supplement table 3. We can see that the identified significant risk factors are consistent with those displayed in Table 2. This indicates that the identified risk factors were not affected by the unbalance of number of patients with history of coronary heart disease in two groups.

### 3.5. Logistic Regression Nomodiagram (Nomogram)

**Figure 1:**
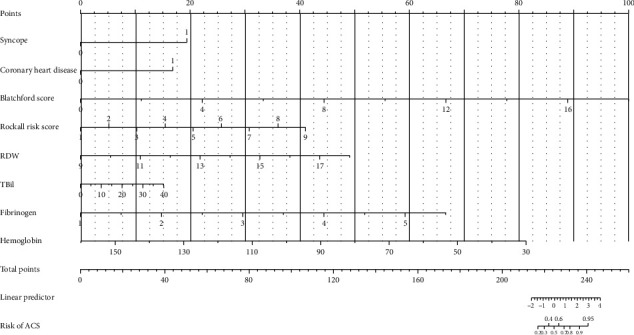
The nomogram of the multivariate logistic regression model. A nomogram can simplify statistical predictive models into a single numerical estimate of the probability of ACS occurrence in the form of a graph. In a nomogram, the value of each patient is located on the axis of variables, and a straight line is drawn upward to determine the point value of each variable. For example, a patient can get 0 point if he has no syncope syndrome and get about 45 points if his Blatchford score is 8. Finally, the total point of this patient is obtained by summing the point values identified on the scale for each variable in the model. If the total point of this patient is 220, the probability of ACS occurrence can be predicted as 20%~30% by a perpendicular line extending downward to “Risk of ACS” axis from 220 on the “Total points” axis.

The C-index of the logistic regression is 0.989, which indicates the model has good discrimination performance. This model is simplified into the nomogram graph (see Figure 1). The prognostic nomogram that integrated all significant independent factors from multivariate analysis for ACS in UIGB patients is shown in Figure 1.

### 3.6. Random Forest Analysis

**Figure 2:**
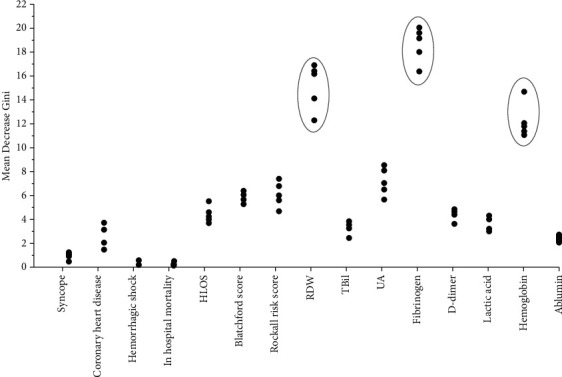
The Mean Decrease Gini of risk factors associated with ACS based on random forest analysis with fivefold cross-validation. The MDG represents the weight of each risk factor by univariate analysis associated with ACS in this model. With MDG sequencing, fibrinogen, RDW, and hemoglobin were ranked the top three risk factors associated with ACS in each of the fivefold cross-validation.

We performed random forest analysis to assess potential factors extracted by univariate analysis associated with ACS. We applied fivefold cross-validation to implement this analysis. The results showed that the classification accuracy is 96.2%,96.8%,98.4%,92.0%, and 94.0%, respectively. The Mean Decrease Gini (MDG) represents the weight of each risk factor in this model. With MDG sequencing, we observed that fibrinogen, RDW, and hemoglobin ranked the top three risk factors associated with ACS in each of the fivefold cross-validation (see Figure 2).

### 3.7. ROC Analysis

**Figure 3:**
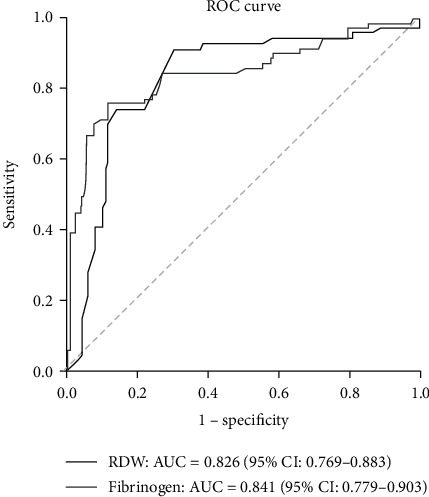
ROC analysis for RDW and fibrinogen associated with ACS. Fibrinogen (AUC = 0.841, 95% CI: 0.779-0.903) and RDW (AUC = 0.826, 95% CI: 0.769-0.883) were strong predictors for ACS through the ROC curve.

**Figure 4:**
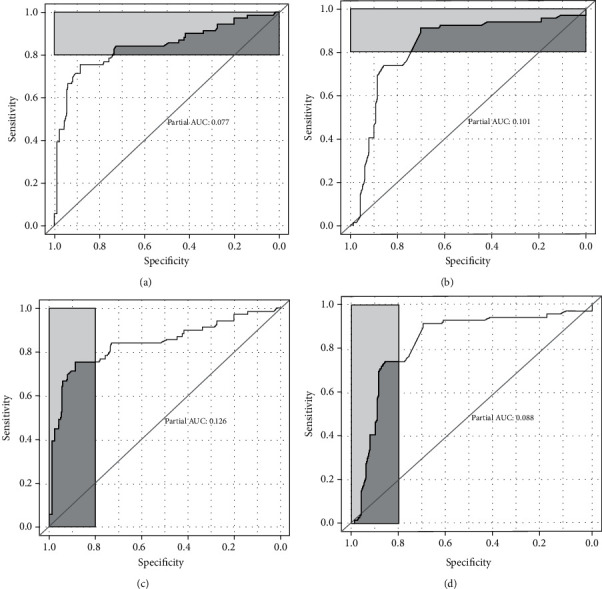
pAUC for fibrinogen and RDW was associated with ACS. Partial AUC (pAUC) is derived from focusing on a partial sensitivity or specificity: (a) pAUC of fibrinogen according to sensitivity > 80%, pAUC = 0.077; (b) pAUC of RDW according to sensitivity > 80%, pAUC = 0.101; (c) pAUC of fibrinogen according to specificity > 80%, pAUC = 0.126; (d) pAUC of RDW according to specificity > 80%, pAUC = 0.088.

Two factors, fibrinogen (AUC = 0.841, 95% CI: 0.779-0.903) and RDW (AUC = 0.826, 95% CI: 0.769-0.883), obtain good discrimination performance (see Figure 3). There were no significant differences between these two factors in AUC (*P* = 0.696). We also performed partial AUC (pAUC) analysis for these two factors focused on sensitivity > 80% and specificity > 80%, respectively. According to sensitivity > 80%, the pAUC of fibrinogen and RDW are 0.077 and 0.101, respectively (Figures 4(a) and 4(b)), and there is no significant difference (*P* = 0.326). However, according to specificity > 80%, the pAUC of fibrinogen is much higher than that of RDW (0.126 vs. 0.088, *P* = 0.018) (Figures 4(c) and 4(d)).

## 4. Discussion

The mortality of UGIB is closely related to ACS after gastrointestinal hemorrhage. ACS is often overlooked in severe gastrointestinal bleeding as its symptoms and signs are often covered up by serious bleeding in the digestive tract. Gastrointestinal bleeding, especially massive hemorrhage, can cause hypovolemia, hemodynamic damage, and myocardial hypoperfusion, leading to the occurrence of ACS. When Aschenbrenner [9] reported the case of UGIB complicated with acute myocardial infarction (AMI) since 1934, people pay attention to the incidence of UGIB combined with ACS [10, 11]. Yet, risk factors for UGIB combined with ACS have not been identified. Previous studies have shown that the presence of some risk factors suggests that UGIB is prone to ACS combination: Bhatti et al. [10] found that multiple risk factors of coronary artery disease and history of coronary artery disease were risk factors for UGIB with AMI, and there was an increase of in-hospital mortality rate after ACS concurrence. A randomized controlled trial involving more than 50,000 patients with coronary artery disease [6] showed that UGIB doubles the risk for AMI in patients with coronary artery disease, particularly in women and patients younger than 65. Emenike et al. [12] expounded that the risk of AMI in patients with gastrointestinal bleeding in the intensive care units increased by three times in patients over 65 years old, while the risk of AMI in patients with two or more coronary heart disease risk factors increased by nine times.

In order to further study the related risk factors of UGIB combined with ACS, this study adopted the method of propensity score matching to analyze selected clinical indicators, with the purpose of predicting the occurrence of ACS after UGIB to reduce mortality rate. This retrospective study in patients with UIGB showed that fibrinogen and RDW are important risk factors for ACS; hemoglobin plays a protective role in the likelihood of occurrence of ACS in these patients. The risk for ACS in the setting of UIGB is higher in patients with syncope, a history of coronary heart disease, and high serum bilirubin, while Glasgow-Blatchford score and Rockall score systems predict the prognosis of them.

Red blood cell volume distribution width (RDW) is a simple, fast, and convenient way to reflect the size difference of red blood cells. It is a quantitative index of about 100,000 red blood cell volume variations in blood circulation measured by a fully automatic hematology analyzer in a few seconds. Increased RDW has clinical significance for evaluating the clinical outcomes and severity of various pathological conditions, including cardiovascular disease, cerebrovascular disease, sepsis, tumor, leukemia, renal insufficiency, and respiratory disease [13–16], and it is considered that the dynamic change of RDW is a strong predictor of patient death [17]. Studies have shown that RDW is an independent risk factor or predictor of the occurrence, development, and prognosis of coronary heart disease [18, 19], and RDW at the upper limit of the normal range is associated with a significant increase in the incidence and mortality of ACS [20]. However, the mechanism of RDW elevation in ACS patients still lacks systematic and in-depth scientific research. At present, the most recognized mechanisms are as follows: (1) activation of the neuroendocrine system. In patients with ACS, the function of cardiac pump is decreased, and compensatory neurohumoral mechanism is triggered, which leads to the increase of hormone levels including angiotensin, norepinephrine, vasopressin in the blood, and the production of erythropoietin, therefore accelerating the production of erythropoiesis and increase of the number of immature red blood cells, which result in the increase of cell heterogeneity finally [21, 22]. (2) Inflammatory effects: ACS patients are commonly accompanied by partial or systemic inflammatory reactions. The inflammatory factors such as IL-1, IL-6, and TNF-*α* could generate the disorder of iron utilization and the decrease of the responsiveness of the bone marrow to erythropoietin, suppress the effect of antiapoptosis, and promote cell maturation [23], which in turn leads to increase in the number of immature cells released into the peripheral circulation, thus boosting the red blood cell heterogeneity. (3) Abnormal red blood cells might participate in the development of myocardial fibrosis through inflammatory amplification, which will cause decreased oxygenation in many organs including cardiomyocytes, thus affecting the cardiovascular system to varying degrees. In severe cases, ischemia and organ failure may occur [24, 25].

Felker et al. [26] first proposed in 2007 that elevated RDW was an independent predictor of prognosis of chronic heart failure, which was independently associated with all-cause death in patients with chronic heart failure. They analyzed data of 2679 patients with chronic heart failure and found that an increase in RDW had the highest correlation with prognosis (death or readmission for heart failure) from indicators of routine blood test. In 2008, Tonelli et al. [27] shifted the research scope for the first time from heart failure to stable coronary heart disease. Through analysis, they found that RDW level of such patients was correlated with Major Adverse Cardiovascular Events (MACEs) and all-cause death: for every 1% increase in RDW, the risk of all-cause death increased by 14%. In 2009, Lippi et al. [28] evaluated 456 patients with ACS and 1848 patients with non-ACS before reaching a conclusion that the median RDW level was much higher in ACS patients than in the non-ACS control group; if RDW was combined with troponin T, the sensitivity of diagnosis of ACS can be increased to 99%.In addition to the diagnosis of ACS, Nabais et al. [29] put forward the effect of RDW on the prognosis of ACS by following up 1796 ACS patients: increased RDW was independently positively correlated with death or recurrence of myocardial infarction within six months of onset. Ma et al. [30] also conducted a single-center cohort study with a large sample and found that RDW increased gradually with the progressive progression of coronary heart disease in patients with coronary atherosclerotic heart disease, suggesting that high RDW may be a risk factor for AMI or ACS. This study also presents the same result that high RDW is a strong risk factor for the combination of ACS in UGIB patients. Lee et al. [31] analyzed 1596 patients with AMI for one year of follow-up and found that the occurrence of MACEs within one year was closely related to the level of RDW at the onset. Following that, Vaya et al. [32] studied the correlation between cardiovascular disease (CVD) events recurrence and RDW levels in myocardial infarction (MI) patients and found that high RDW levels increased the risk of CVD events by six times. Skjelbakken et al. [33] reported a positive association between RDW and AMI risk in the general population, with an increase of ±13% for every 1% increase in RDW. In addition, Azab et al. [34] conducted a study on patients with NSTEMI. Results from a four-year follow-up found that the risk of death increased by 1.1 times when RWD level increased by 1 unit, indicating that RDW could predict the long-term all-cause mortality in NSTEMI patients. Gul et al. [35] studied 310 patients with UA or NSTEMI and found that RDW was a significant predictor of worse outcomes in these patients at the time of hospitalization, while mortality was significantly increased in patients with high levels of RDW over the next 3 years. RDW was clearly associated with mortality in ACS patients and high risk of MACEs. A meta-analysis involving 10,410 patients suggested that low RDW was significantly associated with low mortality in ACS [36]. Therefore, RDW can be used as a risk classification tool for monitoring ACS for UGIB patients, and it has the advantages of being relatively economical, convenient, and readily available. Nevertheless, further investigation is needed to evaluate the efficacy and accuracy of RDW in ACS after UGIB.

Fibrinogen is related to the degree of coronary atherosclerosis in ACS patients, which is positively correlated with the plaque burden of coronary atherosclerosis [37], and is an independent factor influencing the severity of coronary artery lesions in ACS patients. This study showed that the increase of fibrinogen was associated with the combination of ACS after UGIB, which could be used as a clinical indicator for monitoring ACS. Fibrinogen is a coagulation/inflammation marker that can be easily detected [38]. In ACS patients, fibrinogen is higher than that in stable coronary heart disease patients or healthy people, while high fibrinogen may predict poor prognosis in patients [39]. Fibrinogen can participate in coronary atherosclerosis process via the following mechanisms: (1) in the process of endothelial injury, fibrinogen can not only stimulate platelet and leukocyte adhesion in the vessel wall and cells and trigger neurotransmitter release, but it also helps adjust the permeability of endothelial cells and promote the migration of endothelial cells therefore causing dysfunction of endothelial cells. (2) Fibrinogen can promote the proliferation of smooth muscle cells and induce chemotaxis of monocytes, while smooth muscle cells and monocytes/macrophages are the main cellular components of coronary atherosclerotic plaques. (3) Fibrinogen is not only involved in the inflammatory reaction process, but it is also an acute phase reactant [40]. In acute inflammatory responses, interleukin-6 (IL-6), interleukin-1*β* (IL-1*β*), and glucocorticoids could further induce fibrinogen expression, leading to cascade amplification and promoting inflammatory responses [41]. (4) Fibrinogen is mainly involved in blood viscosity, platelet aggregation, and fibrin formation to regulate coagulation activation, while coagulation activation and fibrinolysis or fibrinogen oxidation may exacerbate existing coronary heart disease. The results of multivariate logistic regression analysis in this study showed that fibrinogen was a relevant influencing factor for the occurrence of ACS after UGIB, which was consistent with previous research results [42, 43]. The increase of fibrinogen level was independently correlated with the medium and high syntax score (SXscore) of ACS patients, and the high SXscore indicated that patients with ACS were prone to more severe condition and worse prognosis [44]. A prospective observational cohort study suggested that the fibrinogen to albumin ratio (FAR) could be used to predict MACEs in ACS patients undergoing percutaneous transluminal intervention (PCI) [45]. A single-center prospective cohort study revealed that fibrinogen was positively correlated with the entire ACS population. Elevated baseline fibrinogen level is likely to be an important and independent indicator for MACEs after PCI, especially in patients with diabetes. However, as follow-up time prolongs, the baseline fibrinogen level gradually lost the ability to predict MACEs [46]. Fibrinogen is considered a potential risk factor for the prognosis of patients with ACS, and the fibrinogen level at admission in Chinese ACS patients is independently associated with the risk of death [47].

This study suggested that syncope occurrence and hemoglobin decrease were possible risk factors for ACS after UGIB. Syncope in UGIB patients is mostly a result of hypovolemic shock, indicating severe bleeding. However, hemoglobin is a protective factor for UGIB, and low hemoglobin also indicates hypovolemia and severe gastrointestinal bleeding. Both of these factors can lead to aggravation of myocardial ischemia, thus more likely affecting the occurrence and prognosis of ACS [48, 49]. In 1994, Schwertner et al. [50] accidentally found that low bilirubin levels may be associated with the occurrence of coronary heart disease, thus proposing that low serum bilirubin may be a new risk factor for coronary heart disease. A general perception believes that bilirubin has the effect of antilipid peroxidation and scavenging free radical damage. This endogenous antioxidant could prevent the occurrence of ACS. Serum total bilirubin (STBL) is negatively correlated with coronary plaque vulnerability, and the reduction of STBL may be an important factor in the formation of coronary atherosclerotic plaque [51]. STBL level is associated with MACEs in ACS patients [52, 53]. A meta-analysis suggested that higher STBL significantly improved the prognosis of ASC. STBL was an important factor in the long-term prognosis of vascular disease prevention and could be used as a predictor of vascular-related diseases [54], whereas studies on the relationship between STBL and the risk of coronary heart disease are not entirely consistent. Huang et al. [55] reported that initial STBL level in patients with AMI was positively correlated with short-term mortality. This study also showed that STBL of ACS patients was slightly higher than that of the control group, which was consistent with Huang's conclusion. The conclusion of this study was likely to be affected by the number of samples and the specific time of STBL detection, so the STBL level of UGIB combined with ACS needs to be further evaluated.

In clinical practice, there are various scoring methods to predict death of UGIB patients, including primarily Rockall scores, Glasgow Blatchford score, AIMS65 score, and Charlson scores [56–58]. In consideration of both accuracy and convenience, Chinese doctors adopted mostly Glasgow Blatchford score and Rockall score in clinical practice. Studies have shown that Glasgow Blatchford score was superior to Rockall score in predicting clinical outcomes of UGIB patients [59], but some other scholars have found that the two scores had the same predictive ability for UGIB patients while Blatchford was superior to Rockall scores in predicting whether patients need blood transfusion in clinical treatment [58, 60]. Rockall scores were most strongly correlated with duration of admission and with rebleeding requiring surgery [58]. An international multicenter prospective study illustrated that the Glasgow Blatchford score had high accuracy in predicting the need for hospital intervention or death, with a score ≤ 1 appearing to be the optimal threshold for guiding patients to outpatient treatment [61]. Other studies have found that complete Rockall score was more suitable for prediction of one-month mortality, while Glasgow Blatchford score system worked better for prediction of other outcomes like transfusion need, intensive care unit admission rate, and endoscopic intervention rate [62]. This study exposed in multiple factors analysis that both Rockall score and Glasgow Blatchford score were risk factors for UGIB patients combined with ACS, which indicated both score systems had a certain significance when predicting UGIB patients combined with ACS. In consequence, Rockall score and Glasgow Blatchford score should be performed as much as possible for hospitalized UGIB patients, to timely predict the risk of blood transfusion, rebleeding, and death, as well as the risk of ACS.

To some extent, this study has some limitations. The bleeding volume of UGIB and the diagnostic methods of ACS were not analyzed. However, somehow, these factors did not significantly affect the results considering the purpose of this study. What is more, this study is a single-center, retrospective study with a relatively small sample size, which may affect the bias of research results. Consequently, the conclusion of this study needs to be confirmed by further large-scale prospective studies.

## 5. Conclusion

This study showed that the likelihoods to have UGIB combined with ACS was higher during the same period when hospitalized. Patients should be highly vigilant to the occurrence of ACS in circumstances of increased fibrinogen and RDW, combination with basic heart disease, syncope, significant decrease of hemoglobin, and high levels of total bilirubin. UGIB patients should also perform Rockall score and Glasgow Blatchford score to timely predict the risk of UGIB combined with ACS.

## Figures and Tables

**Table 1:** Characteristics and single-factor analysis of the ACS group and control group (*n* (%), mean ± SD).

Variables	ACS group (*n* = 69)	Control group (*n* = 276)	Statistical magnitude	*P* value
*Demographic parameters*				
Age (years)	68.52 ± 7.58	68.91 ± 7.67	-0.345	0.730
Sex (male)	54 (78.3)	208 (75.4)	0.254	0.614
BMI	25.24 ± 2.83	24.95 ± 3.73	-1.131	0.258
*Clinical manifestations*				
Haematemesis	34 (49.3)	113 (40.9)	1.568	0.211
Melena	67 (97.1)	267 (96.7)	0.023	0.878
Syncope	20 (29.0)	30 (10.9)	14.619	≤0.001
Hemorrhagic shock	31 (44.9)	74 (26.8)	8.577	0.003
*Combined diseases*				
Hypertension	39 (56.5)	159 (57.6)	0.027	0.870
Coronary heart disease	44 (63.8)	65 (23.6)	41.311	≤0.001
Atrial fibrillation	3 (4.3)	9 (3.3)	0.194	0.659
Cerebrovascular disease	21 (30.4)	95 (34.4)	0.393	0.531
Liver cirrhosis	6 (8.7)	24 (8.7)	0.000	1.000
Chronic renal disease	10 (14.5)	40 (14.5)	0.000	1.000
Rheumatic disease	3 (4.3)	7 (2.5)	0.644	0.422
History of gastrointestinal bleeding	23 (33.3)	82 (29.7)	0.342	0.559
Type 2 diabetes	26 (37.7)	110 (39.9)	0.109	0.741
*Lifestyles*				
Smoking	33 (47.8)	140 (50.7)	0.186	0.667
Drinking	25 (36.2)	92 (33.3)	0.207	0.649
*Drug combination*				
Aspirin	31 (44.9)	105 (38.0)	1.095	0.295
Clopidogrel	18 (26.1)	45 (16.3)	3.539	0.060
Other antiplatelet drugs	1 (1.4)	5 (1.8)	0.042	0.837
Anticoagulants	1 (1.4)	5 (1.8)	0.042	0.837
Glucocorticoid	2 (2.9)	2 (0.7)	2.276	0.131
*Etiology of UGIB*				
Peptic ulcer	55 (79.7)	210 (76.1)	0.407	0.524
Malignant tumor	5 (7.2)	15 (5.4)	0.332	0.565
Esophageal and gastric varices	6 (8.7)	24 (8.7)	0.000	1.000
Mallory-Weiss syndrome	0 (0)	4 (1.4)	1.012	0.314
Acute gastric mucosal lesion	1 (1.4)	10 (3.6)	0.845	0.358
Oesophagitis	1 (1.4)	5 (1.8)	0.042	0.837
Anastomositis	1 (1.4)	2 (0.7)	0.336	0.562
*Interventions*				
Mechanical ventilation	2 (2.9)	5 (1.8)	0.328	0.567
Transfusion	39 (56.5)	128 (46.4)	2.275	0.131
CPR	3 (4.3)	10 (3.6)	0.080	0.777
*Hospital indexes*				
In-hospital mortality	7 (10.1)	5 (1.8)	11.418	0.001
HLOS	18.10 ± 3.72	15.12 ± 4.85	-5.680	≤0.001
*Clinical scores*				
Blatchford score	12.93 ± 1.95	10.06 ± 2.91	-7.253	≤0.001
Rockall score	6.84 ± 1.07	4.96 ± 1.76	-8.099	≤0.001
*Laboratory parameters*				
Hemoglobin (g/L)	68.10 ± 6.26	85.28 ± 18.95	12.566	≤0.001
RDW (%)	13.83 ± 1.61	12.02 ± 1.48	-8.390	≤0.001
Blood platelet (10^9^/L)	184.94 ± 51.58	196.28 ± 83.37	1.420	0.157
ALT (IU/L)	29.77 ± 27.10	31.87 ± 36.49	-0.721	0.471
TBil (*μ*mol/L)	14.81 ± 5.75	13.10 ± 7.45	-3.377	0.001
Creatinine (*μ*mol/L)	81.13 ± 30.06	89.28 ± 62.30	-1.413	0.158
BUN (mmol/L)	13.25 ± 5.70	14.11 ± 6.56	-0.769	0.442
UA (*μ*mol/L)	415.48 ± 95.58	323.50 ± 107.96	-6.770	≤0.001
Albumin (g/L)	33.09 ± 4.09	35.01 ± 4.19	-2.934	0.003
INR	1.09 ± 0.26	1.08 ± 0.18	-0.055	0.956
Fibrinogen(g/L)	3.98 ± 0.86	2.89 ± 0.64	-9.870	≤0.001
D-dimer (*μ*g/mL)	1.61 ± 1.48	1.46 ± 1.75	-2.910	0.004
PaO_2_ (mmHg)	83.76 ± 3.28	84.77 ± 8.04	-1.855	0.064
PaCO_2_ (mmHg)	35.66 ± 3.73	36.26 ± 3.89	-1.348	0.178
Lactic acid (mmol/L)	1.82 ± 0.72	1.45 ± 0.71	-5.178	≤0.001

Abbreviation notes: BMI: body mass index; CPR: cardiopulmonary resuscitation; HLOS: hospital length of stay; RDW: red cell distribution width; ALT: alanine aminotransferase; TBil: total bilirubin; BUN: blood urea nitrogen; UA: uric acid; INR: international normalized ratio; PaO_2_: oxygen partial pressure; PaCO_2_: partial pressure of carbon dioxide.

**Table 2:** Logistic regression analysis of relative indexes for ACS in UGIB patients adjusted by the history of coronary heart disease.

Index	Regression coefficient	Standard error	Wald	*P* value	OR value	95% CI
Syncope	4.830	1.427	11.459	0.001	125.162	7.639-2050.687
Coronary heart disease	4.932	1.496	10.871	0.001	138.594	7.389-2599.474
Hemorrhagic shock	-2.568	1.466	3.068	0.080	0.077	0.004-1.358
In hospital mortality	-4.801	3.332	2.075	0.150	0.008	0.000-5.646
HLOS	0.077	0.079	0.958	0.328	1.081	0.925-1.262
Blatchford score	1.299	0.357	13.215	≤0.001	3.665	1.820-7.383
Rockall risk score	1.236	0.430	8.240	0.004	3.441	1.480-8.000
RDW	1.415	0.402	12.406	≤0.001	4.117	1.873-9.048
TBil	0.118	0.059	3.994	0.046	1.125	1.002-1.263
UA	0.006	0.005	1.616	0.204	1.006	0.997-1.016
Fibrinogen	3.100	0.739	17.595	≤0.001	22.188	5.214-94.428
D-dimer	0.437	0.229	3.636	0.057	1.549	0.988-2.428
Lactic acid	-0.409	0.587	0.485	0.86	0.665	0.210-2.098
Hemoglobin	-0.162	0.051	10.189	0.001	0.851	0.770-0.939
Albumin	-0.250	0.150	2.752	0.097	0.779	0.580-1.046

Abbreviation notes: HLOS: hospital length of stay; RDW: red cell distribution width; TBil: total bilirubin; UA: uric acid.

## Data Availability

All data generated or analyzed during this study are included in the supplementary file. Please see supplement, matched data.

## Supplementary Materials

Supplementary Materials In this study, a total of 69 UGIB patients who had developed ACS were selected in ACS group, and 607 UGIB patients without ACS were selected in the control group. Supplement table 1: the observation indices in both groups are shown. Propensity score matching (PSM) was performed by the ratio of 1 : 4 between the ACS and non-ACS groups. PSM variables were age and gender. Supplement table 2: there were 69 cases in the ACS group and 276 cases in the control group after PSM, and then, the age and gender of the two groups were balanced. To validate the identified risk factors for ACS in UGIB, we additionally performed multiple logistic regression analysis without adjust history of coronary heart disease. Supplement table 3: the results for the multiple logistic regression analysis are shown (Supplement tables). The matched data provided the data after PSM. The qualitative data were represented by 0 and 1, with 0 for “None” and 1 for “Yes.” The quantitative data were presented with concrete data.
