# Supplementary material for: Risk Factors for Acute Coronary Syndrome in Upper Gastrointestinal Bleeding Patients
Source: Gastroenterol Res Pract. 2021 Mar 8;2021:8816805. doi: 10.1155/2021/8816805 (PMC7964100; doi:10.1155/2021/8816805)
Supplement: Supplementary Materials — In this study, a total of 69 UGIB patients who had developed ACS were selected in ACS group, and 607 UGIB patients without ACS were selected in the control group. Supplement table 1: the observation indices in both groups are shown. Propensity score matching (PSM) was performed by the ratio of 1 : 4 between the ACS and non-ACS groups. PSM variables were age and gender. Supplement table 2: there were 69 cases in the ACS group and 276 cases in the control group after PSM, and then, the age and gender of the two groups were balanced. To validate the identified risk factors for ACS in UGIB, we additionally performed multiple logistic regression analysis without adjust history of coronary heart disease. Supplement table 3: the results for the multiple logistic regression analysis are shown (Supplement tables). The matched data provided the data after PSM. The qualitative data were represented by 0 and 1, with 0 for “None” and 1 for “Yes.” The quantitative data were presented with concrete data. [file 8816805.f1.docx]

Supplement table 1 Characteristics of ACS group and control group with UGIB [n (%), mean ± SD]

| Variables | ACS group(n=69) | control group(n=607) |
| --- | --- | --- |
| **Demographic parameters**  Age(years)  Sex(male) | 68.52±7.58  54(78.3) | 61.34±14.70  413(68.0) |
| BMI | 25.24±2.83 | 25.07±3.44 |
| **Clinical manifestations** |  |  |
| Haematemesis | 34(49.3) | 265(43.7) |
| Melena | 67(97.1) | 587(96.7) |
| Syncope  Hemorrhagic shock  **Combined diseases** | 20(29.0)  31(44.9) | 76(12.5)  152(25.0) |
| Hypertension | 39(56.5) | 341(56.2) |
| Coronary heart disease | 44(63.8) | 132(21.7) |
| Atrial fibrillation | 3(4.3) | 20(3.3) |
| Cerebrovascular disease | 21(30.4) | 153(25.2) |
| Liver cirrhosis | 6(8.7) | 57(9.4) |
| Chronic renal disease | 10(14.5) | 57(9.4) |
| Rheumatic disease | 3(4.3) | 22(3.6) |
| History of gastrointestinal bleeding  Type2 diabetes  **Life styles**  Smoking  Drinking  **Drug combination**  Aspirin  Clopidogrel  Other antiplatelet drugs  Anticoagulants  Glucocorticoid  **Etiology of UGIB**  Peptic ulcer  Malignant tumor  Esophageal and gastric varices  Mallory Weiss syndrome  Acute gastric mucosal lesion  Oesophagitis  Anastomositis  **Interventions**  Mechanical ventilation  Transfusion  CPR  **Hospital indexes**  In hospital Mortality  HLOS  **Clinical scores**  Blatchford score  Rockall risk score  **Laboratory parameters**  Hemoglobin(g/L)  RDW(%)  Blood platelet (10^9^ /l)  ALT(IU/L)  TBil(umol/L)  Creatinine (umol/L)  BUN (mmol/L)  UA (umol/L)  Albumin(g/L)  INR  Fibrinogen (g/L)  D-dimer(ug/ml)  PaO_2_(mmHg)  PaCO_2_(mmHg)  Lactic acid(mmol/L) | 23(33.3)  26(37.7)  33(47.8)  25(36.2)  31(44.9)  18(26.1)  1(1.4)  1(1.4)  2(2.9)  55(79.7)  5(7.2)  6(8.7)  0(0)  1(1.4)  1(1.4)  1(1.4)  2(2.9)  39(56.5)  3(4.3)  7(10.1)  18.10±3.72  12.93±1.95  6.84±1.07  68.10±6.26  13.83±1.61  184.94±51.58  29.77±27.10  14.81±5.75  81.13±30.06  13.25±5.70  415.48±95.58  33.09±4.09  1.09±0.26 3.98±0.86  1.61±1.48  83.76±3.28  35.66±3.73  1.82±0.72 | 171(28.2)  209(34.4)  284(46.8)  209(34.4)  229(37.7)  94(15.5)  11(1.8)  12(2.0)  13(2.1)  459(75.6)  37(6.1)  57(9.4)  9(1.5)  19(3.1)  13(2.1)  6(1.0)  15(2.5)  265(43.7)  20(3.3)  13(2.1)  15.15±4.93  10.00±3.05  4.89±1.80  86.48±18.38  12.09±1.60  199.50±82.18  29.89±30.67  13.28±7.53  81.60±52.15  13.26±6.25  313.33±101.59  35.54±4.02  1.08±0.16  2.90±0.63  1.37±1.65  84.92±7.76  36.08±4.02  1.53±0.79 |

Abbreviation notes:

BMI: body mass index CPR: cardiopulmonary resuscitation HLOS: hospital length of stay

RDW: red cell distribution width ALT: alanine aminotransferase TBil: total bilirubin BUN: blood urea nitrogen UA: uric acid INR: international normalized ratio PaO2: oxygen partial pressure PaCO2: partial pressure of carbon dioxide

Supplement table 2 Comparison of matching variables after PSM

| variable | ACS group (n=69) | Control group (n=276) | t or χ2 | *P* |  |
| --- | --- | --- | --- | --- | --- |
| Age | 68.52±7.58 | 68.91±7.67 | -0.345 | 0.730 |  |
| Male/female | 54/15 | 208/68 | 0.254 | 0.614 |  |

Supplement Table 3 Logistic regression analysis of relative indexes for ACS in UGIB patients without adjust by the history of coronary heart disease

| Index | Regression  Coefficient | Standard  error | Wald | P value | OR value | | 95%CI |
| --- | --- | --- | --- | --- | --- | --- | --- |
| \| Syncope  Hemorrhagic shock  In hospital mortality  HLOS  Blatchford score  Rockall risk score  RDW  TBil  UA  Fibrinogen  D-dimer  Lactic acid  Hemoglobin  Albumin \| \| --- \| | 3.257  0.676  -4.664  0.118  1.126  0.929  1.060  0.122  0.007  2.413  0.257  -0.014  -0.122  -0.123 | 1.009  0.755  2.119  0.071  0.281  0.303  0.257  0.051  0.003  0.530  0.192  0.457  0.034  0.092 | 10.426  0.801  4.844  0.790  16.103  9.394  17.031  5.727  5.193  20.724  1.803  0.001  12.535  1.781 | **0.001**  0.371  0.028  0.095  **≤0.001**  **0.002**  **≤0.001**  **0.017**  0.023  **≤0.001**  0.179  0.976  **≤0.001**  0.182 | 25.976  1.965  0.009  1.125  3.084  2.533  2.887  1.130  1.007  11.166  1.294  0.986  0.885  0.885 | 3.597-187.591  0.448-8.629  0.000-0.600  0.980-1.292  1.779-5.347  1.398-4.589  1.745-4.775  1.022-1.249  1.001-1.014  3.951-31.555  0.988-2.428  0.403-2.415  0.828-0.947  0.739-1.059 | |

Abbreviation notes:

HLOS: hospital length of stay RDW: red cell distribution width TBil: total bilirubin UA: uric acid
